# Spatial distribution of antenatal care utilization and associated factors in Ethiopia: evidence from Ethiopian demographic health surveys

**DOI:** 10.1186/s12884-018-1874-2

**Published:** 2018-06-18

**Authors:** Abraham Yeneneh, Kassahun Alemu, Abel Fekadu Dadi, Atinkut Alamirrew

**Affiliations:** 1 College of Medicine and Health Sciences Institute of Public Health, Department of Health Informatics, University of Gondar, Gondar, Ethiopia; 2 College of Medicine and Health Sciences, Institute of Public Health, Department of Health Informatics, Department of Epidemiology and Biostatistics, University of Gondar, Gondar, Ethiopia

**Keywords:** Antenatal care utilization, Spatial distribution, Ethiopia

## Abstract

**Background:**

Antenatal care (ANC) is one of the components of care to be provided to pregnant women. In Ethiopia, characterizing the spatial distribution of antenatal care utilization is essential to prioritize risk areas where ANC is needed and facilitate interventions. Therefore, this spatial analysis was performed to assess the spatial distribution of ANC utilization between 2000 and 2011 and to identify factors associated with ANC utilization in Ethiopia.

**Methods:**

A total of 23,179 women who had a live birth in the five years preceding the surveys were included in the study. The spatial data were created in ArcGIS10.1 for each study clusters. The Bernoulli model was used by applying Kulldorff methods using the SaTScan™ software to analyze the purely spatial clusters of ANC utilization. Multiple logistic regression analysis was used to identify predictors affecting ANC utilization.

**Results:**

ANC utilization had spatial variations across the country. Spatial scan statistics identified 49 high performing clusters (LLR = 111.92, *P* < 0.001) in 2000, 51 (LLR = 114.49, *P* < 0.001) in 2005 and, 86 (LLR = 121.53, *P* < 0.001) in 2011. ANC utilization was higher among mothers; with richest wealth quintiles, lowest number of birth order, who are living in urban areas, younger and educated.

**Conclusion:**

These results provide further insight into differences in ANC utilization in the country and highlight high and modest performing clusters. This could enable efficient and timely spatial targeting to improve ANC service up take in Ethiopia.

## Background

Antenatal care refers to the regular medical and nursing care recommended for women during pregnancy to prevent, detect and treat complications [1]. Timely initiation of antenatal care is an opportunity to prevent the direct causes of maternal deaths and reduce fetal and neonatal deaths related to obstetric complications [2].

Globally, about 300,000 women die each year as a result of pregnancy related complications [3], 2.6 million babies are estimated to be stillborn [4], and 4 million newborns die in the neonatal period. In sub-Saharan Africa and other resource-poor settings, maternal mortality remains a major public health problem [3, 5]. Such deaths could be prevented if pregnancy and birth-related complications are managed properly through the expansion and uptake improvement of basic antenatal care service [6].

In Ethiopia, the levels of maternal and infant morbidity and mortality are among the highest in the world [7]. These major problems could be addressed by proper utilization of ANC, skilled delivery and postnatal care services [8]. Geographic accessibility of the services is one of the most important factors associated with utilization of maternity care [9]. Rural populations are particularly disadvantaged as they often lack reliable means of transport [10]. Women who have a higher density of clinics nearby have a lower risk of starting antenatal care late [11].

Understanding the levels and geographical variations of ANC utilization would help policymakers, planners, programmers and partners in the health sector to formulate appropriate strategies and interventions and provide quality reproductive health services. The aim of this study was to assess the spatial distribution and identify factors associated with ANC utilization using evidence from the Ethiopian Health and Demographic Surveys between 2000 and 2011.

## Methods

### Study design and study settings

A repeated cross sectional study design was employed using the Ethiopian Demography and Health Surveys (EDHS) in 2000, 2005, and 2011 to assess spatial pattern of ANC utilization. Ethiopia is located in the horn of Africa. It has a total area of 1,100,000 km^2^ and lies between latitudes 3° and 15°N, and longitudes 33° and 48°E. Ethiopia has been divided into nine ethnic based and politically autonomous regional states and two cities (Addis Ababa and Dire Dawa). The Regions are subdivided into sixty-eight zones, and then further into 817 districts which are further divided into around 16,253 Kebeles (the lowest local administrative units).

### Study population

The source population was all women of childbearing age (15–49 years) in Ethiopia. The study population was all women who had a live birth in the five years preceding the survey just to minimize the recall bias arising from the time relapse. The most recent birth was considered for women with two or more live births during the five-year period. All records related to ANC utilization, which had complete records in all EDHS (EDHS-2000, 2005 and 2011) documents were included in the study.

### Sample size and sampling procedure

A total of 23,179 women (7967 women in 2000, 7304 women in 2005 and, 7909 women in 2011) were included in the study. Weighted values were used because weights restore the representativeness of sampled data. Sample weights were calculated and included in each EDHS recoded file. Data were extracted using STATA version 12.0 and SPSS version 20 software.

A Multi-stage stratified sampling technique was used for all three EDHS. In 2000 and 2005, sampling was based on the Census Enumeration Areas (CEAs) of the 1994 Population and Housing Census of Ethiopia [12]. In 2011, sampling was based on the 2007 National Population and Housing Census of Ethiopia [13].

In 2000 and 2005, a total of 540 (139 urban and 401 rural) CEAs were selected using systematic sampling from all regional states in the country. Then, in 2000, 27 households per CEA and in 2005, 24 to 32 households per CEA were selected systematically. In 2011, a total of 624 CEAs (187 urban and 437 rural) were selected using systematic sampling technique.

### Data collection tools and procedures

ANC data were obtained from central statistics agency (CSA) using www.dhsprogram.com. The web provided the data only for authorized users. Data also comprised the location data (latitude and longitude coordinates).

### Statistical analysis

Descriptive and multivariable logistic regression analyses were carried out using STATA 12. Independent variables were age, birth order, region, education, wealth index, and ANC accessibility (e.g. residence, transport and distance). Both bi and multivariable binary logistic regression models were used to identify factors that affect ANC utilization. Those variables with *P*-value < 0.2 in bi-variable logistic regression model were entered in the multivariable logistic regression model to measure the effect of each variable after adjusting for the effect of other variables using Backward Logistic Regression. Variables with *p*-value < 0.05 were considered as statistically significant.

A Bernoulli-based model was used in which events at particular places were analyzed if women use ANC or not represented by a 0/1 variable. The scan statistics developed by Kulldorff and SaTScan™ software version 9.4 [8] were used to identify the presence of purely spatial low ANC utilization clusters. Scan statistics did scanning gradually across the space to identify the number of observed and expected observations inside the window at each location. The scanning window with the maximum likelihood was the most likely high performing clusters, and a *p*-value was assigned to this cluster. The window centered on each of the several possible grid points positioned throughout the study areas. For each grid point, the radius of the window differed continuously in size from zero to a specified maximum value. ANC utilization clusters centered on one of the grid squares used as spatial units of observation.

Spatial interpolation technique was applied to predict the unsampled/unmeasured value from sampled measurements. In the DHS survey ANC utilization is known for all census enumeration areas, but ANC utilization for other unselected locations in Ethiopia is also of interest. The one with the maximum likelihood comprised the highest performing cluster. The *p*-value was obtained through the Monte Carlo hypothesis testing by comparing the rank of the maximum likelihood from the real datasets with the maximum likelihoods from the random datasets. The number of replications were 999. Secondary clusters were identified in the datasets in addition to the most likely cluster and we ordered them according to their likelihood ratio test statistic. The inferences of secondary clusters were adjusted for more likely clusters in the data using an iterative manner. The maximum cluster size was set at 50% of the population at risk.

## Results

There are 14,642 households and 15,716 eligible women in 2000, 14,645 households and 14,717 eligible women in 2005, and 18,720 households and 17,385 eligible women in 2011. From this, a response rates for the first, second, and third surveys were 97.8% (*n* = 15,370), 95.6% (*n* = 14,069), and 95% (*n* = 16,515), respectively. The actual women included in this study were 7967 in 2000, 7304 in 2005, and 7909 in 2011.

### Socio-demographic characteristics

**Fig. 1:**
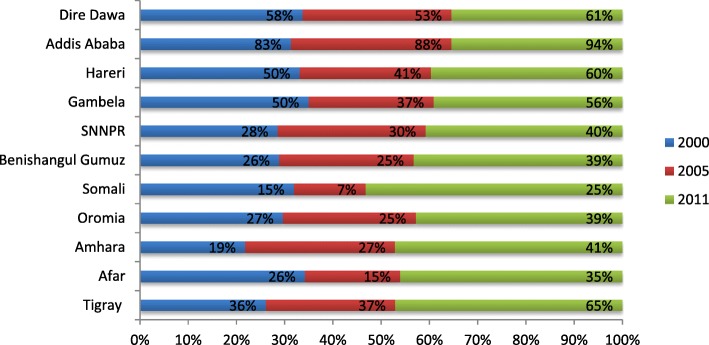
Trends in ANC utilization overtime by regions, in 2000, 2005 and 2011, Ethiopia

Of the total respondents, in 2000, 7061 (88.6%), in 2005, 6673 (91.3%), and in 2011, 6720 (85%) were rural residents. The majority of women ages during birth were between 25 to 29 years old. Women who had 2 to 4 birth order were 3259 (43.8%) in 2000, 3026 (41.4%) in 2005, and 3464 (40.9%) in 2011. In 2000, 6539 (82.0%), in 2005, 5734 (78.5%), and in 2011, 5270 (66.6%) women were uneducated. Of the total women in 2005, 1520 (20.8%) and in 2011 1739 (22.0%) were in the lowest wealth quintile (Table 1). Despite the low coverage of ANC utilization, all regions registered an increasing trend from 2000 to 2011 (Fig. 1).

**Table 1:** Socio-demographic characteristics of women participated on EDHS (2000, 2005, 2011), Ethiopia

	Year					
	2011		2005		2000	
Variables	Frequency (*n* = 7909)	Percent	Frequency (*n* = 7304)	Percent	Frequency (*n* = 7967)	Percent
Place of residence						
Urban	1189	15	633	8.7	906	11.4
Rural	6720	85	6671	91.3	7061	88.6
Age at birth						
15–19	402	5.1	440	6.0	472	5.9
20–24	1608	20.3	1473	20.2	1727	21.6
25–29	2383	30.1	1960	26.8	2021	25.6
30–34	1489	18.8	1427	19.5	1493	18.6
35–39	1239	15.7	1138	15.6	1219	15.3
40–44	572	7.2	578	7.9	706	8.9
45–49	216	2.7	288	4.0	329	4.1
Birth order						
1	1399	17.7	1190	16.3	1362	17.1
2–4	3465	43.8	3024	41.4	3259	40.9
> =5	3045	38.5	3090	42.3	3346	42.0
Level of education						
No education	2506	31.7	5734	78.5	6539	82.1
Primary	3483	44.1	1205	16.5	1003	12.6
Secondary	1068	14.5	325	4.5	400	5.0
Higher	852	10.7	40	0.5	25	0.3
Wealth quintile						
Poorest	1739	22.0	1520	20.8		
Poorer	1696	21.5	1553	21.26		
Middle	1628	20.6	1585	21.71		
Richer	1494	18.8	1451	19.86		
Richest	1352	17.1	1195	16.37		
Region						
Tigray	530	6.7	480	6.6	536	6.7
Affar	78	0.9	68	0.9	85	1.1
Amhara	1991	25.2	1856	25.4	2222	27.8
Oromia	3116	39.4	2723	37.3	3057	38.4
Somali	198	2.5	288	3.9	84	1.1
Benishangul-gum	92	1.2	68	0.9	81	1.0
SNNP	1634	20.7	1631	22.3	1689	21.2
Gambela	31	0.4	23	0.3	22	0.3
Harari	19	0.2	15	0.3	16	0.2
Addis Ababa	193	2.4	128	1.8	148	1.9
Dire Dawa	27	0.4	24	0.3	27	0.3

### Spatial distribution of ANC utilization

Spatial variation was found in ANC utilization at regional levels. Of a total of 7909 women interviewed, 3361 (42.5%) women utilized ANC by skilled health care providers. The highest utilization of ANC was spatially clustered in Addis Ababa. On the other hand, Somali Region was the lowest in ANC utilization (Table 1). Of a total of 7304 women interviewed in 2005, 2016 (27.6%) utilized ANC. Of a total of 7967 women interviewed in 2011, 2127 (26.7%) utilized ANC. Out of these, the highest ANC utilization was in Addis Ababa, Dire Dawa, Hareri and Gambela sequentially, while Amhara and Somali Region showed the lowest ANC utilization (Table 1).

### Spatial pattern of ANC utilization

**Fig. 2:**
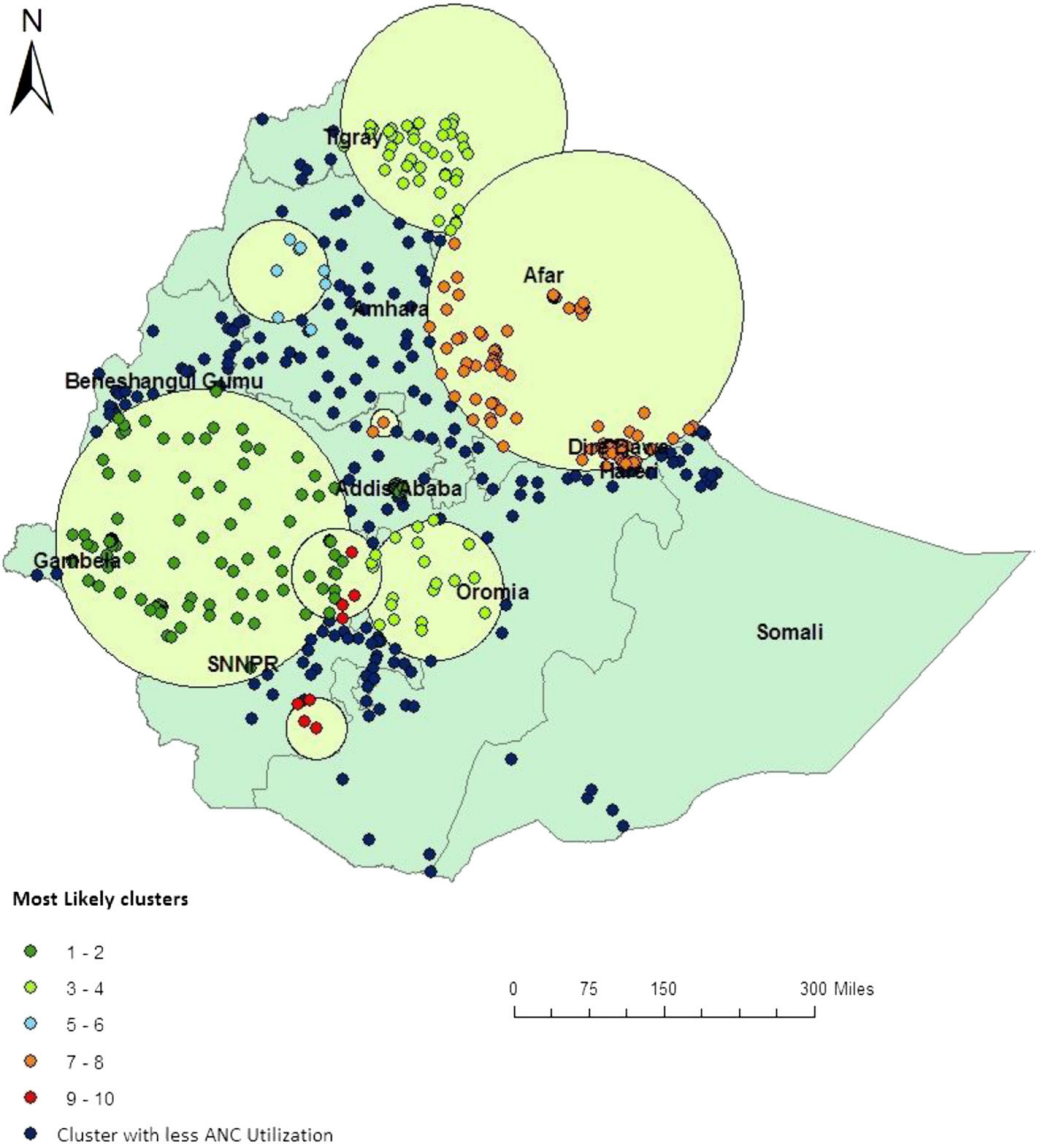
Spatial distribution of ANC utilization in 2000 EDHS

In EDHS 2000, spatial scan statistics identified a total of 145 high and modest performing spatial clusters of ANC utilization. Of these, 49 clusters were high performing clusters) (LLR = 111.92, *P* < 0.001) accounting for 33.7% and 96 clusters were modest performing clusters (LLR = 71.15, *P* < 0.001) accounting for 66.3%. The highest performing clusters of ANC utilization were detected in Addis Ababa, Dire Dawa, Harari, Oromia, Gambela and Tigray regions. The modest ANC utilization clusters were located around Somali and Amhara Regions (Fig. 2).

**Fig. 3:**
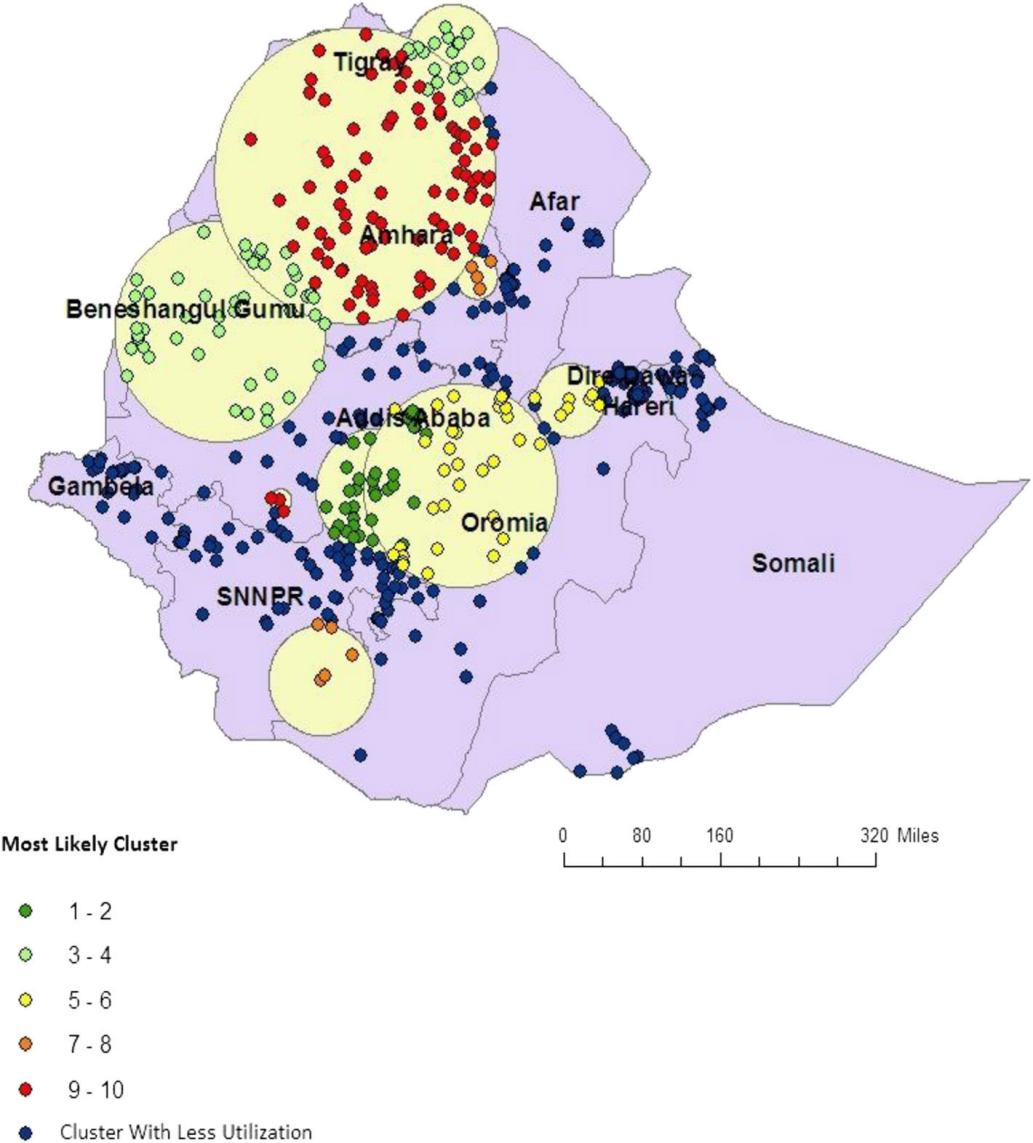
Spatial distribution of ANC utilization in 2005 EDHS

In EDHS 2005, spatial scan statistics identified a total of 78 spatial clusters of ANC utilization. Of these, 51 spatial clusters were high performing (LLR = 114.49, *P* < 0.001) and 27 clusters were modest performing clusters (LLR = 99.04, *P* < 0.001) and accounted 53.9% of the clusters. The high performing clusters of ANC utilization were detected in Addis Ababa, Dire Dawa, Harari, some part of SNNP, Benishangul Gumuz and Tigray regions. The modest ANC utilization clusters were detected in Somali and Gambela Regions (Fig. 3).

**Fig. 4:**
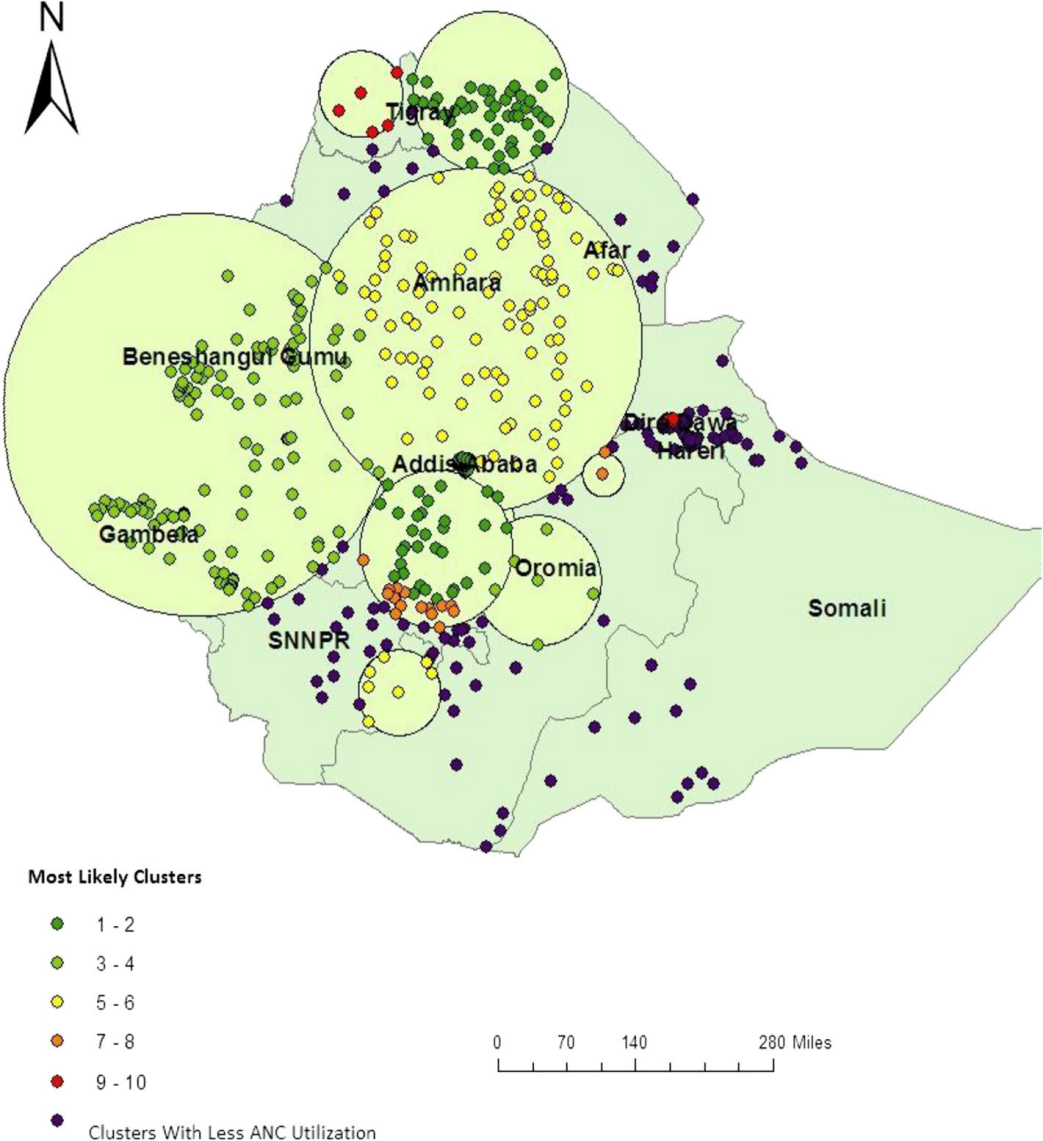
Spatial distribution of ANC utilization in 2011 EDHS

In EDHS-2011, spatial scan statistics identified a total of 136 spatial clusters of ANC utilization. Of these, 86 clusters were high performing clusters (LLR = 121.53, *P* < 0.001) while 50 clusters were modest perfuming clusters (LLR = 61.87, *P* < 0.001). The high performing spatial clusters of ANC utilization were detected in Addis Ababa, Dire Dawa, Harari, Gambela, Benishangul Gumuz and Tigray regions. The modest antenatal care service utilization clusters were mostly located around Somali and some part of Afar Regions (Fig. 4).

### Interpolation using GIS mapping

**Fig. 5:**
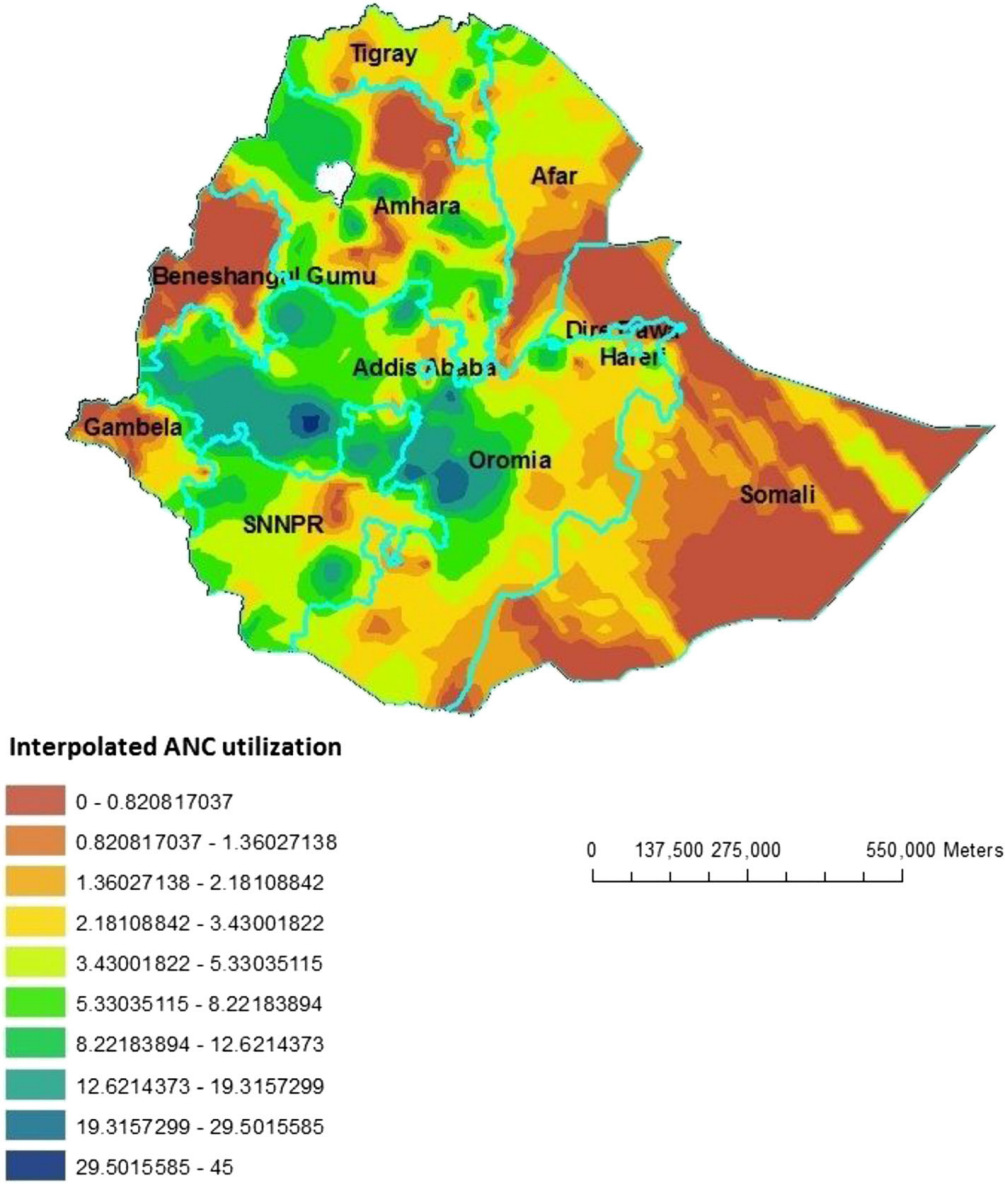
Interpolated special distribution of ANC utilization in 2000, EDHS

**Fig. 6:**
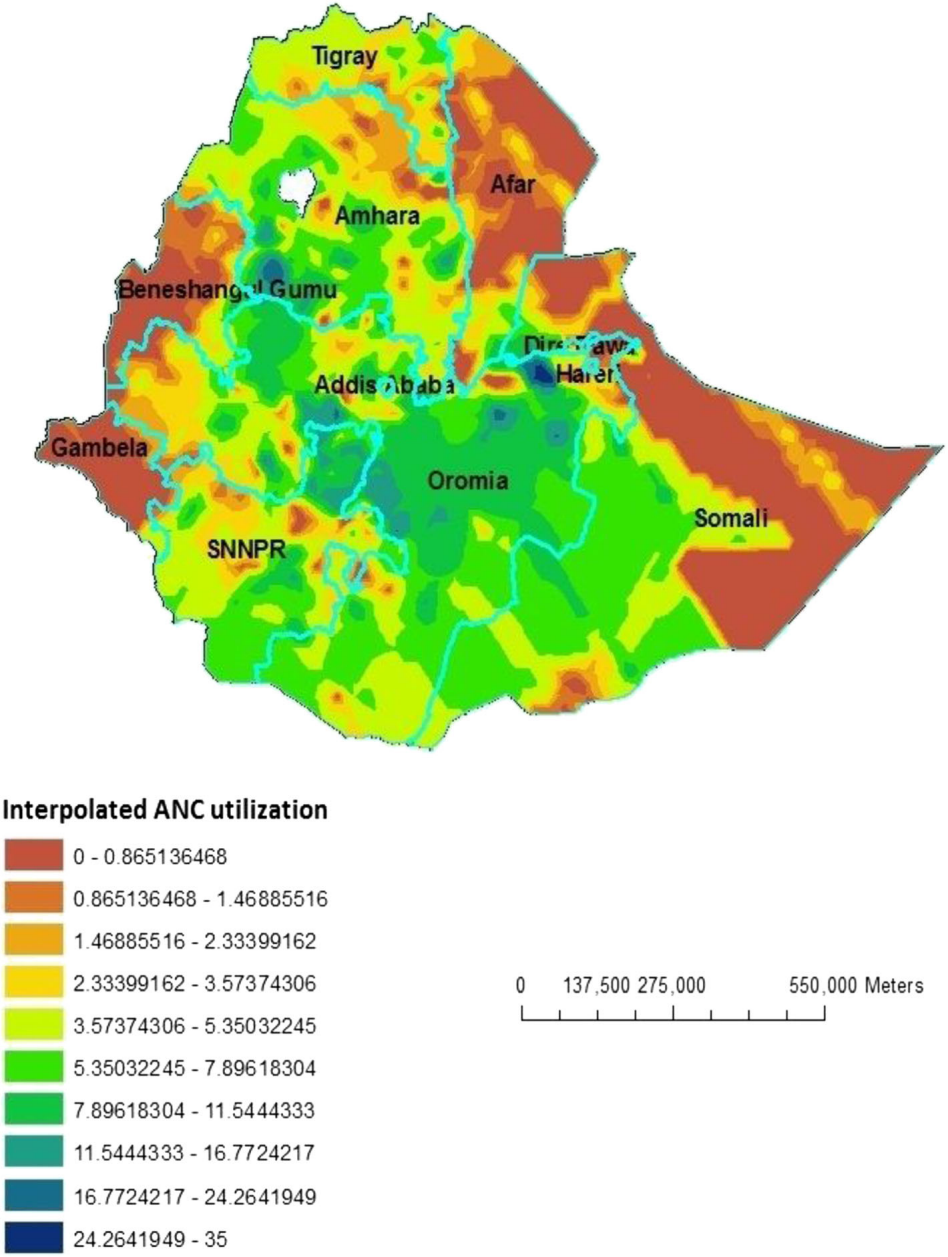
Interpolated special distribution of ANC utilization in 2005, EDHS

**Fig. 7:**
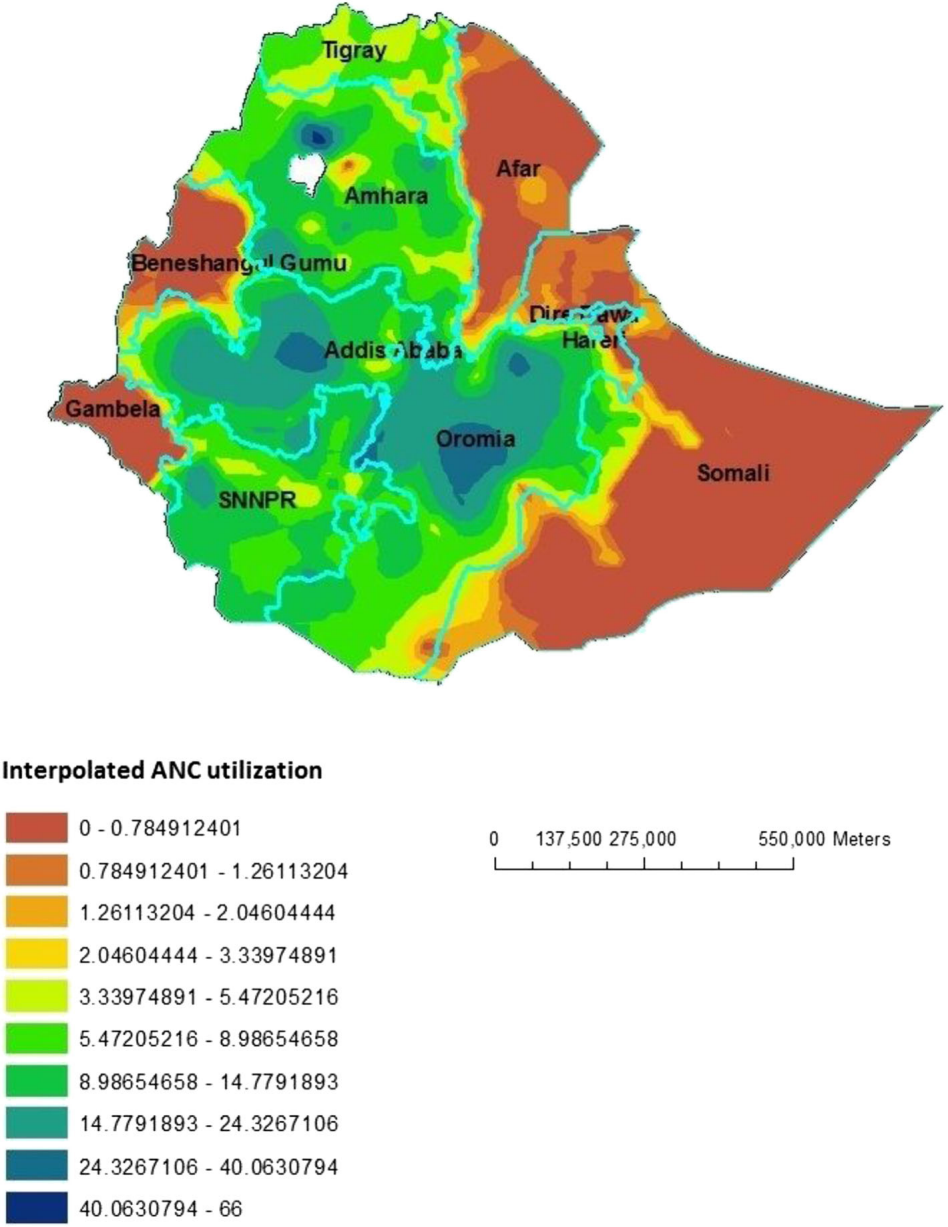
Interpolated special distribution of ANC utilization in 2011, EDHS

Based on EDHS-2000 sampled data, geostatistical analysis predict that highest ANC utilization rates were detected in South-West Oromia region, some Northern part of Oromia and some parts of southern SNNPR. In contrast, relatively low ANC utilization areas were predicted in Beneshangul Gumuz, Western part of Gambela, Northern part of Affar and most part of Somali. Based on EDHS-2005 Geostatistical analysis, high ANC utilization was detected in Southwest part of Amhara, Northeast Oromia and Southeast part of SNNPR. In contrast to this, low ANC utilization places were in the Eastern part of Somali, most part of Affar and Gambela and Western part of Beneshangul Gumuz. From the third EDHS sampled data in 2011, geostatistical analysis predicted that most part of Oromia, Northern Amhara and some part of Western SNNPR contained the highest ANC utilization areas, while most parts of Afar, Somali, Beneshangul Gumuz and Gambela contained modest ANC utilization areas (Figs. 5, 6 and 7).

### Factors associated with ANC utilization for EDHS-2011

After adjusting for different confounding variables, place of residence, age at birth, level of education, region, birth order and wealth status were significantly associated with ANC utilization (Table 2).

**Table 2:** Women’s socio-demographic characteristics and its association with utilization of antenatal care service 2011, Ethiopia

Variable	Received ANC		Crude odds ratio (95% CI)	Adjusted odds ratio (95% CI)
	Yes	No		
Place of residence				
Urban	893	297	5.21 (4.52–5.99)	1.55 (1.22–1.97)
Rural	2471	4248	1	1
Age at birth				
15–19	160	242	0.82 (0.66–1.02)	0.80 (0.61–1.06)
20–24	787	822	1.19 (1.05–1.36)	1.18 (1.00–1.38)
25–29	1059	1324	1	1
30–34	577	912	0.79 (0.69–0.90)	0.80 (0.68–0.95)
35–39	512	727	0.88 (0.77–1.01)	0.94 (0.78–1.13)
40–44	207	364	0.71 (0.59–1.86)	0.83 (0.65–1.06)
45–49	63	153	0.52 (0.38–0.70)	0.59 (0.41–0.85)
Birth order				
1	753	646	1.53 (1.35–1.74)	1.34 (1.13–1.59)
2–4	1495	1969	1	1
> =5	1117	1929	0.76 (0.69–0.84)	1.01 (0.87–1.18)
Mothers Education				
No education	888	1618	0.45 (0.40–0.49)	0.31 (0.23–0.56)
Primary	1916	1567	1	1
Secondary	943	125	6.17 (5.06–7.52)	4.67 (4.02–6.65)
Higher	796	56	11.62 (8.79–15.36)	7.44 (6.98–9.01)
Wealth quintile				
Poorest	436	1303	1	1
Poorer	593	1103	1.607 (1.39–1.86)	1.54 (1.31–1.81)
Middle	616	1012	1.82 (1.57–2.11)	1.86 (1.58–2.19)
Richer	704	790	2.66 (2.30–3.09)	3.01 (2.54–3.56)
Richest	1016	336	9.07 (7.69–10.68)	5.79 (4.52–7.40)
Region				
Tigray	328	202	2.76 (2.28–3.34)	2.85 (2.28–3.57)
Affar	27	51	0.82 (0.51–1.31)	0.86 (0.49–1.51)
Amhara	794	1197	1.01 (0.90–1.14)	1.02 (0.89–1.17)
Oromia	1232	1884	1	1
Somali	50	148	0.52 (0.37–0.72)	0.37 (0.25–0.55)
Benishangul	37	55	1.03 (0.67–1.56)	1.02 (0.63–1.65)
SNNP	660	974	1.04 (0.92–1.17)	1.13 (0.98–1.31)
Gambela	18	13	2.03 (0.99–4.15)	2.03 (0.86–4.75)
Harari	11	8	2.22 (0.89–5.52)	1.02 (0.35–2.97)
Addis Ababa	180	13	21.54 (12.16–38.16)	4.00 (2.14–7.48)
Dire Dawa	16	11	2.29 (1.05–5.03)	1.14 (0.44–2.94)

Women who lived in urban areas were 1.55 times more likely to utilize ANC than women living in rural areas [AOR = 1.55; 95% CI; 1.22–1.97]. As the wealth of the women increases the odds of utilizing ANC also increases. The odds of ANC utilization was 0.31 [AOR = 0.31; 95%CI; 0.23–0.56] times lower among non-educated group, 4.67 [AOR = 4.67; 95%CI; 4.02–6.65] times higher among mothers with secondary education and 7.44 [AOR = 7.44; 95%CI; 6.98–9.01] times higher among mothers with higher education as compared to mothers with primary level of education. Women’s in age group of 30–34 and 45–49 were 20% [AOR = 0.80; 95%CI; 0.68–0.95] and 41% [AOR = 0.59; 95%CI; 0.41–0.85] less likely to utilize ANC as compared to women in the age group of 25–29. Mothers whose their pregnancy is for the first time were 1.34 times more likely to utilize ANC [AOR = 1.34; 95%CI; 1.13–1.59]. Compared to women living in Oromia region, those living in Tigray [AOR = 2.85; 95%CI; 2.28–3.57] and Addis Ababa [AOR = 4.00, 95%CI; 2.14–7.48) were more likely to utilize ANC (Table 2).

## Discussion

SaTScan and GIS-based spatial statistical techniques provide an opportunity to clarify and identify antenatal care utilization within a country and help future investigations into factors responsible for increased antenatal care utilization. Geographic visualization of ANC utilization using these tools was instrumental to understand highest and modest utilization level and target interventions in high-risk areas. This study has provided a visually powerful analysis of spatial variation in antenatal care utilization among women in Ethiopia.

ANC utilization rate has substantially increased from 26.7% in 2000 to 27.6% in 2005 and 42.5% in 2011 in Ethiopia. High variations, however, are observed between rural and urban areas. This variation may be because of proximity of the health facility in the village and the geographical distribution of ANC clinics. These reasons are consistent with findings of another study in Brooklyn, US [11]. In all surveys, women from urban areas had less numbers than women from rural areas (11.4% versus 88.6%, in 2000, 8.7% versus 91.3% in 2005, and 15% versus 85% in 2011, respectively. There is also variation in antenatal care utilization across the country’s regions. Highest antenatal care utilization rates were reported in Addis Ababa, Dire Dawa, Harari, whereas comparatively modest utilization was reported in Somali regions and Affar. These regions are relatively urban in which health facilities are more accessible and women are more aware of maternal health services. The multivariable model also revealed consistent finding in which Tigray and Addis Ababa were the places where the highest ANC utilization is recorded and Somali is found to be the place where small number of mothers got ANC as compared to the biggest region (Oromia). These reasons are consistent with the findings of another study in Ethiopia [14].

Another factor that affected ANC utilization was the income of the mother, the odds of ANC utilization being higher among women in the richest wealth quintile. Even though, ANC is currently under exempted service in health facilities in Ethiopia, it was not like this before and women should pay to get the service. In addition, income would also affect health seeking behavior of the mother in which poor mothers had low health seeking behavior [15, 16].

Age of the mother and birth order are the other predictor variables reported to have an association with ANC utilization. Relatively younger women and whose pregnancy is for their first time were more likely to attend ANC service as compared to older and multiparous women. Similar studies have also reported an indirect relationship between parity and maternal services; women’s of high parity tend to use ANC less [17, 18]. This could be explained by low to no experience about the current pregnancy will make the new mother to have some sort of fear of risks associated with her pregnancy that make her more curios to have frequent ANC visits. The perception that they had better knowledge about pregnancy complications would be another factor that explains low ANC uptake by mothers who had more number of children as compared to new mothers [19].

Like previous studies [17–19], in the current analysis, educated women were more likely to utilize ANC as compared to those relatively educated. It is true that education enhances the health seeking behavior of the mother, it also make the mother to actively involved in different knowledge enhancement activities like reading materials, service promotions, and peer discussions. It is also believed that more educated mothers would tend to give better care and concern for their health and their fetus and they are also more likely to strive to know benefits of the service and associated pregnancy complications.

## Conclusion

This study identified spatial clusters of ANC utilization in Somalia Region with low utilization rates and Addis Ababa with the highest utilization rate. Richest wealth quintiles, lowest number of birth order, educated, living in urban areas and being younger increased the likelihood of utilizing ANC. These results provide further insights into identifying the true picture of ANC utilization clusters in the country and enable efficient and timely spatial targeting factors triggering low utilization of antenatal care.

## Abbreviations

ANC: Antenatal care
AOR: Adjusted Odds ratio
CSA: Central statistics agency
EDHS: Ethiopian demographic and health survey
IRB: Institutional Review Board
SNNPR: Southern Nations and Nationality and peoples region
SPSS: Statistical package for social sciences
